# Mowing Submerged Macrophytes in Shallow Lakes with Alternative Stable States: Battling the Good Guys?

**DOI:** 10.1007/s00267-016-0811-2

**Published:** 2017-01-02

**Authors:** Jan J. Kuiper, Michiel J. J. M. Verhofstad, Evelien L. M. Louwers, Elisabeth S. Bakker, Robert J. Brederveld, Luuk P. A. van Gerven, Annette B. G. Janssen, Jeroen J. M. de Klein, Wolf M. Mooij

**Affiliations:** 1 Department of Aquatic Ecology, Netherlands Institute of Ecology, P.O. Box 50, Wageningen, 6700 AB The Netherlands; 2 Aquatic Ecology and Water Quality Management Group, Department of Environmental Sciences, Wageningen University, P.O. Box 47, Wageningen, 6700 AA The Netherlands; 3 Witteveen+Bos, P.O. Box 233, Deventer, 7400 AV The Netherlands; 4 Ecology & Biodiversity, Institute of Environmental Biology, Utrecht University, P.O. Box 80.084, 3508 TB Utrecht, The Netherlands; 5 Royal HaskoningDHV, P.O. Box 1132, 3800 BC Amersfoort, The Netherlands

**Keywords:** Model, Aquatic plant, Harvesting, Phosphorus, Resilience, Ecosystem services

## Abstract

Submerged macrophytes play an important role in maintaining good water quality in shallow lakes. Yet extensive stands easily interfere with various services provided by these lakes, and harvesting is increasingly applied as a management measure. Because shallow lakes may possess alternative stable states over a wide range of environmental conditions, designing a successful mowing strategy is challenging, given the important role of macrophytes in stabilizing the clear water state. In this study, the integrated ecosystem model PCLake is used to explore the consequences of mowing, in terms of reducing nuisance and ecosystem stability, for a wide range of external nutrient loadings, mowing intensities and timings. *Elodea* is used as a model species. Additionally, we use PCLake to estimate how much phosphorus is removed with the harvested biomass, and evaluate the long-term effect of harvesting. Our model indicates that mowing can temporarily reduce nuisance caused by submerged plants in the first weeks after cutting, particularly when external nutrient loading is fairly low. The risk of instigating a regime shift can be tempered by mowing halfway the growing season when the resilience of the system is highest, as our model showed. Up to half of the phosphorus entering the system can potentially be removed along with the harvested biomass. As a result, prolonged mowing can prevent an oligo—to mesotrophic lake from becoming eutrophic to a certain extent, as our model shows that the critical nutrient loading, where the lake shifts to the turbid phytoplankton-dominated state, can be slightly increased.

## Introduction

Shallow lake ecosystems depend on the presence of submerged aquatic plants (macrophytes) for good water quality and high biodiversity (Heimans and Thijsse 1895; Carpenter and Lodge 1986; Jeppesen et al. 1998). There is a positive feedback between aquatic plants and water clarity, through which the plants enhance their own growing conditions (Van Donk and Van de Bund 2002; Scheffer 2004). Such self-stabilizing mechanism causes a tendency of the system to resist changes in external environmental conditions, i.e. it promotes a clear water state within the context of alternative stable states in lakes (Scheffer 2004).

During the second half of the twentieth century, submerged macrophytes disappeared from many shallow lakes in temperate regions because of external nutrient loading from mainly anthropogenic sources (Gulati and Van Donk 2002; Körner 2002). Lakes switched from a clear-water state, dominated by macrophytes, to a turbid-water state with few plants, prone to harmful cyanobacterial blooms (Scheffer et al. 1993; Carpenter et al. 1999). For many years since, tremendous management effort has been devoted to the restoration of aquatic plant communities, mainly through the reduction of external nutrient loading, especially phosphorus (P) (Cullen and Forsberg 1988; Jeppesen et al. 2005; Hilt et al. 2006). Although lakes in the turbid state may also be resilient to changes in external environmental conditions (Hosper 1998), reduction of external nutrient loading is effective in the long run (Jeppesen et al. 2005), and many of the impacted lakes have recovered or are now recovering to a clear-water state with submerged macrophytes (Sondergaard and Moss 1998; Gulati and Van Donk 2002).

Almost inevitable, the return of aquatic plants is accompanied by nuisance caused by these plants (e.g. van Donk 1990). The nutrient availability in restored lakes is generally still rather high, which in combination with improved light conditions allows for rampant growth of rooted macrophytes (Lamers et al. 2012). These dense stands of aquatic plants cause nuisance to bathers and swimmers, which generally dislike the touch of plants and because invertebrates living on the macrophytes may cause itches and rash of the human skin (Van Donk 1990). Dense stands can also cause nuisance for fisherman as lines easily get stuck and because a high macrophyte cover can have a negative effect on fish abundance (Bickel and Closs 2009). Moreover, dense stands can impair (recreational) boat traffic and can decrease lakefront property values. In fact, many functions and ecosystem services may be impacted by the presence of plants (e.g. Van Nes et al. 1999; Anderson 2003). As a result, current management practices are more and more focusing on the reduction of aquatic plants, even though the re-establishment of an aquatic plant community is still considered a prerequisite for the long-term success of lake restoration measures (Van Nes et al. 2002). In many rapidly developing countries nuisance growth of aquatic plants is also readily apparent (Van Ginkel 2011). There, the increased availability of nutrients stimulates plant growth in precedence of a regime shift to a phytoplankton dominated state—a part of eutrophication which also occurred in the temperate lakes before the submerged macrophytes disappeared en mass during the last century (Hasler 1947).

A common human response to excessive growth of submerged macrophytes is mechanical cutting and harvesting (Hilt et al. 2006; Hussner et al. 2016). However, when lakes have alternative stable states, defining a sustainable mowing regime is challenging, given the important role of macrophytes in stabilizing the clear water state. Theory predicts that when a critical, in practice unknown, amount of vegetation is removed, positive feedbacks propel the system to the turbid state with phytoplankton dominance (Scheffer et al. 1993; Van Nes et al. 2002). When less vegetation is removed, on the other hand, the system may show a swift recovery back to the vegetated equilibrium state, undoing the impact of mowing. Van Nes et al. (2002) applied two dynamic aquatic plant models of different complexity to analyze the response of aquatic plant populations to harvesting and concluded that it may be almost impossible to maintain vegetation biomass at any desired intermediate level. Consequently, Van Nes et al. (1999, 2002) suggest it may be more fruitful to assign just a few key functions to entire lakes, than to pursue a compromise between conflicting destinations. In most cases however, lake managers do not have the luxury to divide functions over different lakes, for example due to legal obligations, such as the Water Framework Directive (European Union 2000).

A potentially viable option is to aim for a temporal relief of nuisance following a discrete mowing event. When this period of relief coincides with the moment users are relying on the services provided by the lake, mowing can be convenient despite eventual recovery to the vegetated equilibrium state. Van Nes et al. (2002) did not consider the temporal aspects of mowing in their plant modeling study, as they assumed continuous cutting strategies for simplicity. Yet it remains a tall order for water quality managers to estimate the amount of plant volume that can be safely removed, and predict the period of relief of nuisance after mowing. The numerous field and laboratory studies that have investigated the response of macrophytes and phytoplankton to harvesting (e.g. Engel 1990; Nichols and Lathrop 1994; Barrat-Segretain and Amoros 1996; Morris et al. 2003; Bal et al. 2006; Morris et al. 2006) did not bring general applicable insights as the results were ambiguous. Moreover, lake managers in NW Europe often lack experience as submerged macrophytes were missing for a long time, while formal decision support schemes are basically absent (Hilt et al. 2006). We argue that there is a need for an integrated analysis to obtain a better understanding of the general consequences of plant removal in relation to trophic state and ecosystem resilience.

In this research we use a comprehensive dynamic ecosystem model—PCLake—to study the effect of mowing on shallow lake ecosystems with alternative stable states. This model describes the main nutrient and food web dynamics of a non-stratifying shallow lake in response to eutrophication and re-oligotrophication (Janse and van Liere 1995; Janse 1997), including many feedback mechanisms and processes that have been associated with plants and alternative stable states in lakes. PCLake is frequently used by scientist and water quality managers, mainly in the Netherlands and Denmark, to analyze the complex dynamics of shallow lake ecosystems and to evaluate the effectiveness of potential restoration measures (e.g. Van Liere and Janse 1992; Janse et al. 1993; Janse et al. 1998; Nielsen et al. 2014; Trolle et al. 2014). The model has been calibrated with data from more than 40 temperate shallow lakes located in the Netherlands, Belgium and Ireland (Janse et al. 2010). The aim of this calibration exercise was to obtain a best overall fit for the whole set of lakes, rather than achieving an optimal fit for one specific lake at the expense of others. As a result, the model has a fairly wide geographic applicability and is suitable for generalized studies on temperate shallow lakes (Janse et al. 2010). Hence, PCLake provides a consistent framework that can be used to study how alternative stable states come about, and how they affect ecosystem functioning and ecosystem management. For example, Janse et al. (2008) used the model to study how general lake features, such as depth, fetch and sediment type determine the resilience of shallow lakes to external nutrient loading. Likewise, PCLake has been used to evaluate the importance of rising temperatures (Mooij et al. 2007, 2009), littoral-pelagic coupling (Sollie et al. 2008), allochthonous particulate organic matter (Lischke et al. 2014), tube-dwelling invertebrates (Hölker et al. 2015) and herbivory by birds (Van Altena et al. 2016).

We designed our study to cover several important aspects of mowing that are relevant to ecosystem managers. Firstly, we evaluate how the impact of mowing depends on the trophic status of the lake (i.e. external nutrient loading), mowing intensity and timing of mowing during the growing season. We express the effect of mowing both in terms of remaining plant cover, and in terms of days without nuisance caused either by macrophytes or cyanobacteria. This exercise also allows us to evaluate under which conditions mechanical cutting of macrophytes results in a critical regime shift to the alternative turbid state. Secondly, we use the model to obtain quantitative estimations of the amount of P that can be removed from the system via harvesting of macrophytes. Removal of P may help to remediate eutrophication effects in the lake, and potentially can be recovered for sustainable reuse. Finally, we explore the long term impacts of mowing to analyze whether mowing is a measure that also can be applied to help prevent undesired eutrophication effects in shallow lakes.

## Methods

### Model Description

#### General Features

PCLake consists of a number of coupled ordinary differential equations and auxiliary equations which describe the most important biotic and abiotic components of both the water column and the sediment top-layer of a non-stratifying shallow lake (Janse 1997). By putting equal emphasis on the biotic and abiotic components, the model is unique in its kind (Janssen et al. 2015). Primary producers are represented by submerged macrophtyes and three groups of phytoplankton (diatoms, green algae and cyanobacteria). The food web is completed by detrivorous macrozoobenthos, zooplankton, zooplanktivorous fish, benthivorous fish and piscivorous fish. The abiotic components in the sediment and in the water column are detritus, inorganic material, dissolved phosphorus, ammonium and nitrate. All organic components are modeled in dry-weight (DW), nitrogen (N) and phosphorus (P), and hence the nutrient-to-dry-weight ratios of the organic components are variable. Internal fluxes of nutrients between the sediment layer and the pelagic zone, including internal loading, are accounted for and modeled dynamically. Processes such as diffusion, adsorption, burial, sedimentation and resuspension are included (see Bryhn and Hakanson 2007 for details). The main inputs to the model are: dimensions (depth and fetch), water inflow, nutrient loading, particulate loading, temperature, irradiation and sediment characteristics. PCLake has been calibrated following a beyasian approach to parameter estimation and uncertinty analysis (Aldenberg et al. 1995; Janse et al. 2010). The calibration focussed on higher level variables that are of interest to water quality mangers, including chlorophyll-*a*, Secchi depth, vegetation cover and nutrient concentrations in the water column (Janse et al. 2010). In a recent multi-model ensemble study using an independent dataset, PCLake came out as the most accurate model out of a set of three tested aquatic ecosystem models (Trolle et al. 2014). Although PCLake has mainly been applied to temperate lakes in NW Europe, successful case studies in Mediterranean Greece (Mellios et al. 2015) and Subtropical China (Kong et al. 2016) suggest that the model may also be of value outside the temperate zone. A full description of the model is presented by Janse (2005). A schematic overview of PCLake is presented in Online Resource 1.

#### Alternative Stable States

The PCLake model shows a nonlinear response to changing nutrient loadings, similar to examples studied in the field (Janse 1997). Lakes with a low external nutrient loading are in the clear-water macrophyte-dominated state with low chlorophyll-*a* concentrations. Lakes that receive a high external nutrient input reside in a turbid phytoplankton dominated state. In between, a fairly abrupt shift between the contrasting states takes place. The *critical nutrient loading* for a shift from a clear to a turbid state during eutrophication (CNL_eu_) is at a much higher value than the critical nutrient loading where the reverse switch takes place, back to clear conditions during re-oligotrophication (CNL_oligo_). Hence, at intermediate loading levels both the clear-water state and the turbid water state can exist as alternative stable states and the prevalent state depends on the foregoing conditions—a phenomenon known as hysteresis. Between the critical nutrient loading values, strong perturbations, such as discrete mowing events, may instigate a regime shift from one state to the other (Janse et al. 2008). Classical alternative stable states theory predicts that a lake is more vulnerable to disturbances closer to a tipping point, while the time it takes to recover from a perturbation increases (Van Nes and Scheffer 2007). Previous analyses with PCLake indicated that alternative stable states are most likely to occur in lakes that are shallow (<4 m depth) and have a relatively small fetch (<3000 m) (Janse et al. 2008).

#### Macrophytes

**Fig. 1:**
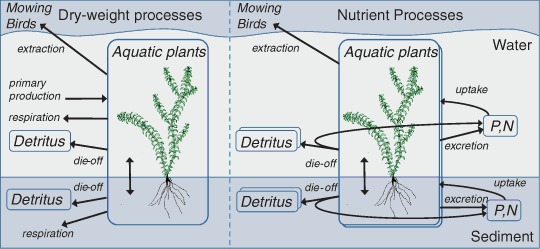
Basic processes of the aquatic plants in PCLake. The modeled processes are nutrient uptake, production, respiration and nutrient excretion, mortality, grazing by birds and mowing. The nutrient processes are modeled both in phosphorus and nitrogen. Herbivory by birds was not considered in this study. The figure is adapted from Janse (2005)

The submerged macrophytes in PCLake represent Waterweeds in general (*Elodea* spp.). Waterweed species are non-native yet widespread in NW Europe and they are often among the first macrophytes to return after restoration measures have been taken (Heimans and Thijsse 1895; Perrow et al. 1997; Pot and ter Heerdt 2014; Immers et al. 2015). They are documented to cause nuisance by their mass development and are subject to mowing management (Hilt et al. 2006; Zehnsdorf et al. 2015). In PCLake, the growth of the submerged macrophytes (Fig. 1) is dependent on nutrient availability, temperature and under-water light availability. Plants take up phosphate, ammonium, and nitrate from both water column and soil pore water to achieve optimal P:biomass and N:biomass ratios (Droop 1974; Madsen and Cedergreen 2002; Angelstein and Schubert 2008; Baldy et al. 2015; Christiansen et al. 2016). Ammonium is preferred, but when the ammonium concentration is low, the plants switch to nitrate uptake. The available light for primary production forms a gradient with depth (Lambert–Beer’s law) and is controlled by the light intensity at the water surface, which is set by a seasonal sine curve (based on long-term averages for Dutch solar irradiance), and by the light attenuation by the plants themselves (self-shading), phytoplankton, detritus and inorganic matter in the water column as well as background extinction. It is assumed that the growing season starts when a critical spring water temperature (9 °C) is reached. This happens in mid-April, given the long-term averaged seasonal water temperature in Dutch lakes. The growing season ends half September onwards, when part of the above-ground biomass is allocated to the below ground biomass, and the mortality of the plants is raised for 2 weeks such that 30% of the original biomass survives, i.e. the over-wintering parts.

The submerged macrophytes are involved in several positive feedbacks with water clarity that have been linked to the emergence of alternative stable states in shallow lakes (Sondergaard and Moss 1998; Scheffer 1999; Horppila and Nurminen 2003; Janse et al. 2008). For example, they are able to suppress phytoplankton growth by being strong competitors for nutrients while having a relatively low light extinction coefficient. Moreover, they provide shelter for phytoplankton grazing zooplankton, and reduce the resuspension caused by wind and benthivorous fish. Furthermore, vegetation promotes growing conditions for piscivorous fish which exert top-down pressure on zooplanktivorous fish. Finally, aquatic plants have the potential to lower the total amount of available nitrogen in the system by promoting denitrification.

A mowing function is available in PCLake, which requires defining a date when the mowing event takes place, the duration of the mowing event and a mowing intensity (i.e. fraction of the biomass that is removed). The mowing intensity is independent of the duration of the mowing event: a natural logarithm is used to calculate the amount of biomass that is removed per day: *h* = *-*ln(1.0–*f*)/p*V, where *h* is the harvested biomass (g m^−2 ^day^−1^), *f* is the intensity (-), *p* is the duration (days) and *V* is the total aquatic plant biomass in the lake (g m^−2^). We applied a 'clean' mowing strategy throughout this study, whereby all biomass is removed from the lake. We did briefly consider potentially harmful side effects of mowing, including enhanced resuspension and incomplete removal of plant material from the water column, but present these findings as an appendix as they did not affect the conclusions of our main analyses (see Online Resource 2).

#### Implementation

We used default parameter settings describing a lake that is representative for many shallow lakes in the temperate zone, with a mean depth of 2 m, a 1000 m fetch, a water inflow of 20 mm day^−1^ (100 day residence time), a lightly clayish soil (30% dry matter, of which 10% organic matter, and 10% lutum), no infiltration or seepage and no surrounding wetland area (c.f. Janse et al. 2010). The N:P ratio of the external nutrient loading was set at 13, i.e. the estimated average N:P ratio for agricultural runoff in the Netherlands (Wolf et al. 2003). In this set-up, the calculated CNL_eu_ and CNL_oligo_ values are 1.6 and 0.9 mg P m^−2^ day^−1^ respectively. To run simulations we used a C++ compiled version of the PCLake model called from GRIND for MATLAB (Mooij et al. 2014).

### Model Simulations

#### Nutrient Loading, Mowing Intensity and Timing

In this study on the impact of mowing on the lake we varied three independent variables of the model that can be controlled by lake managers: (1) external nutrient loading, (2) mowing intensity and (3) timing of the mowing. We first focused on the interplay between the first two. We simulated different combinations of external P loading, ranging from 0.7 to 1.7 in steps of 0.05 (mg m^−2 ^day^−1^), and mowing intensity, ranging from 0 to 0.9 in steps of 0.1 (−). We did not consider P loadings above 1.7 mg m^−2^ day^−1^ as the modeled lake then resides in the turbid water state without macrophytes. Each simulation was started from a clear water state and we ran the model for 20 years before starting the mowing procedure to ensure the lake to be in (seasonal) equilibrium. Note that internal nutrient loading in PCLake is not an independent variable, and by running the model 20 years we achieve that the internal loading in the system associates with the corresponding levels of external P loading. The initialization period was followed by three succeeding years where a mowing event took place. We considered 3 years to include the effect of mowing on the biomass in the next year (Kimbel and Carpenter 1981). Each of the mowing years comprised one discrete mowing event, taking place on July 1st. This is in compliance with the guidelines provided by Rijkswaterstaat, responsible for the management of the main waterways and water systems in the Netherlands, who discourage mowing during the avian breeding season (Rijkswaterstaat 2012). The duration of the mowing event (*p*) was kept at the default value of 10 days in all of these and subsequent cases. Next, we repeated the foregoing simulations, but this time focusing on different combinations of mowing intensity and timing. Again the mowing intensity ranged from 0 to 0.9 in steps of 0.1, while the mowing dates ranged from June 1st to September 1st in steps of 7 days. We performed this analysis for three different nutrient loading settings (0.8, 1.1, and 1.4 g P m^−2^ day^−1^, respectively).

To evaluate the effects of the mowing actions we analyzed the summer average (June 10th–September 15th) vegetation cover and cyanobacterial chlorophyll-*a* concentration in the final year of the simulations. In the model, the vegetation cover increases linearly with the dry weight (DW) of submerged plants until 200 g DW m^−2^ is reached and the cover is 100%. Also, we calculated the days with nuisance during the peak of the holiday season (beginning of July until the end of August) caused by either submerged water plants or cyanobacteria. We presumed that water plants cause nuisance when they cover more than 40% of the area (Gettys et al. 2014). For the cyanobacteria, we followed the Dutch cyanobacteria protocol and took 12.5 mg m^−3^ cyano-chlorophyll as a limit above which nuisance occurs (National Water Overleg 2012). Short-time human exposure to concentrations higher than this value can cause skin rashes or gastrointestinal sickness, and this risk should be communicated to bathing guests.

Additionally, we zoomed in on one intermediate nutrient loading (1.3 mg P m^−2^ day^−1^) and present the within-season dynamics of the vegetation cover and chlorophyll-*a* in response to several different mowing intensities, to also obtain a more detailed view on the dynamics of the lake.

#### Nutrient Removal by Harvesting

We kept track of the amount of P stored in aquatic plant biomass harvested from the system in the final (third) year of mowing, to evaluate the potential to impoverish the lake. The amount of P removed from the system via harvesting provides an indication of the P that can potentially be recovered for reuse. In addition, we calculated the relative removal of P, that is, the ratio of P in the harvested biomass to the total amount of P added to the system via external loading. The relative removal thus allows to assess the extent to which harvesting may contribute to the closing of the P cycle.

#### Prolonged Mowing and the Resilience to Nutrient Loading

We used PCLake to analyze whether harvesting of macrophytes has the potential to forestall eutrophication effects in the long run. More precisely, we analyzed how repeated annual harvesting changes the CNL_eu_ of the lake, that is, the amount of external nutrient loading the lake can withstand without switching to a phytoplankton-dominated turbid state. Following Janse et al. (2008), we calculated CNL_eu_ values for different combinations of mowing intensity and timing, for which we took the same ranges as presented in the foregoing analysis. For each combination the model was evaluated for P loading rates ranging from 0.1 to 4 mg P m^−2^ day^−1^ in steps of 0.1. Each simulation started with a clear and oligotrophic lake. The summer average Secchi depth (m) after 20 years was used to evaluate the state of the lake, to determine which P loading is the CNL­_eu_. Previous analyses have shown that the ratio of Secchi depth to lake depth is a suitable response variable to determine the CNL_eu_ (c.f. Witteveen+Bos 2010; Lischke et al. 2014): above a ratio of 0.5 the lake is defined as clear, while below this ratio the lake is defined as turbid. Mowing took place in each of the 20 years and comprised one discrete mowing event lasting the standard 10 days.

## Results

### Nutrient Loading and Mowing Intensity

**Fig. 2:**
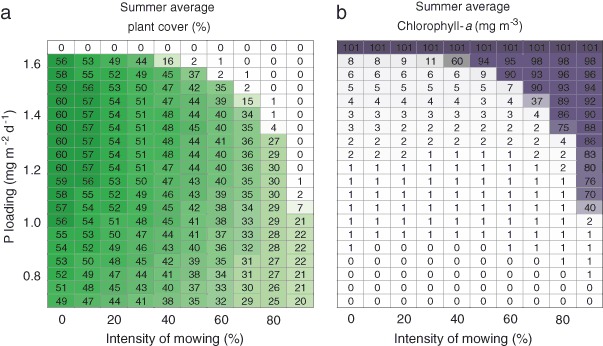
Combined effects of mowing intensity and nutrient loading on summer average plant cover (**a**) and chlorophyll-*a* (**b**) in the final year of the simulations. Mowing starts on July 1^st^

The model shows that the summer average plant cover can be reduced by mowing (Fig. 2a). When external nutrient loading is low, and no alternative equilibrium exists, plant cover shows an almost linear decrease with increasing mowing intensity. At high nutrient loadings however, mowing can trigger a regime shift to an alternative state with high phytoplankton concentrations (Fig. 2b). The mowing intensity that leads to a regime shift shows a nonlinear relationship with nutrient loading; the critical mowing intensity decreases sharply when the external loading approaches the critical nutrient loading (1.61 mg m^−2^ day^−1^). In the vicinity of the critical nutrient loading, a mowing intensity of >30% is sufficient to trigger a collapse when mowing is applied in three succeeding years.

**Fig. 3:**
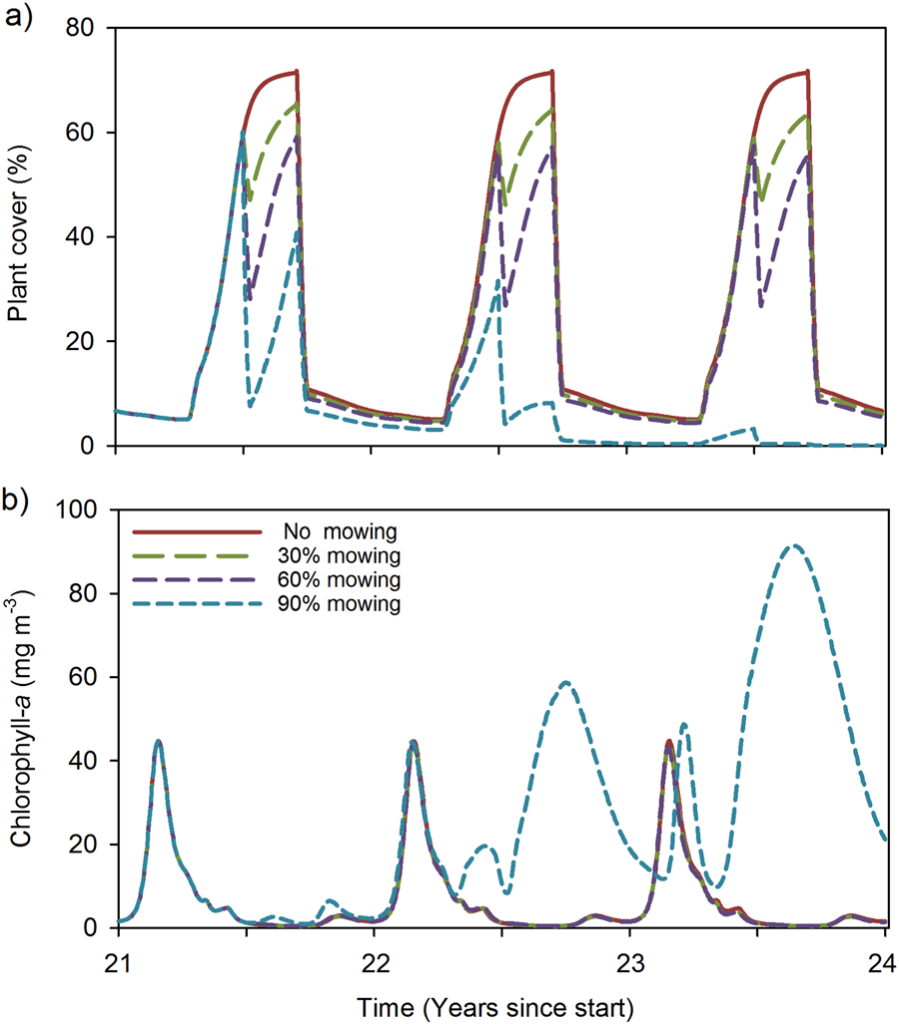
Effects of mowing on July 1st on summer average plant cover (**a**) and chlorophyll-*a* (**b**) in three succeeding years for a lake receiving 1.3 mg P m^−2^ day^−1^

Zooming in on the seasonal dynamics clearly reveals the time window where plant cover is reduced due to mowing lasting for at least several weeks (Fig. 3a). It also shows that, apart from the average plant cover, the maximum plant cover reached during the growing season is also lowered with increasing mowing intensity. A detailed look reveals the importance of considering three succeeding years: the 90% mowing treatment triggers a regime shift, which only becomes apparent in the second and 3rd year, when the plant community collapses and phytoplankton blooms start to occur (Fig. 3b).

**Fig. 4:**
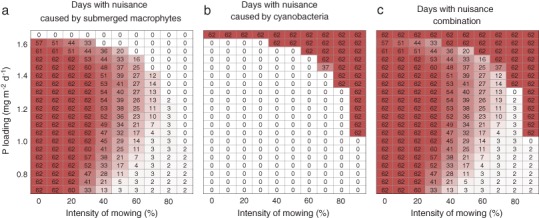
Combined effects of mowing intensity and nutrient loading on days with nuisance caused by aquatic plants (**a**), cyanobacteria (**b**) or both aquatic plants and cyanobacteria (**c**) during July and August (peak of the holiday season in the temperate region) in the final year of the simulations. Mowing starts on July 1^st^

An important question is how the response of the ecosystem to mowing translates to nuisance experienced by lake users. Our approach illustrates that there is a sharp boundary between nuisance caused by macrophytes and nuisance caused by cyanobacteria when the nutrient loading is high (Fig. 4a–c). On the other hand, when the nutrient loading is fairly low (<1 mg m^−2^ day^−1^), mowing can create conditions where hardly any nuisance is experienced during the peak of the summer holiday season (Fig. 4c), given that a substantial fraction of the submerged macrophytes is removed (>50%).

### Timing of Mowing

**Fig. 5:**
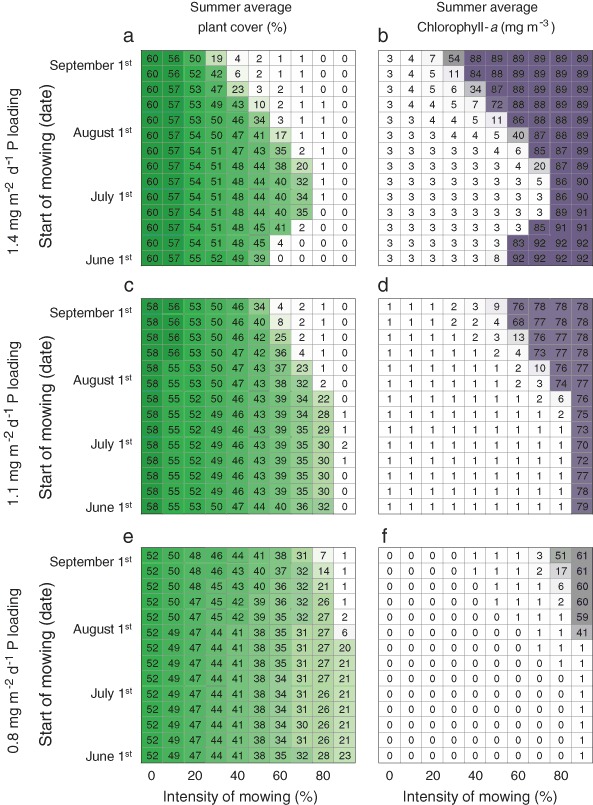
Combined effects of mowing intensity and mowing date on summer average plant cover and chlorophyll-*a* in the final year of the simulations, for three different nutrient loadings: 1.4, 1.1 and 0.8 mg m^−2^ day^−1^, respectively

The impact of harvesting varies during the growing season (Fig. 5), particularly when the external nutrient loading is high (Fig. 5a and b) and the lake is susceptible to a regime shift (Fig. 2a and b). When the nutrient loading is high, the modeled lake is most vulnerable in late summer, when harvesting a fraction of 40% is sufficient to instigate a regime shift to the phytoplankton dominated state. To a somewhat lesser extent, also mowing in early summer eases a shift to the turbid state. The resilience to perturbations of the modeled lake is highest during mid-summer, as up to 80% of the vegetation can be removed, resulting in a halving of the summer average plant cover (Fig. 5a and b). The timing of mowing is not particularly important when the external nutrient loading is low (Fig. 5e and f). Large fractions of the plant biomass can be removed almost the entire growing season without risking a regime shift, allowing to reduce the summer average plant cover up to 40%.

### Nutrient Removal by Mowing

**Fig. 6:**
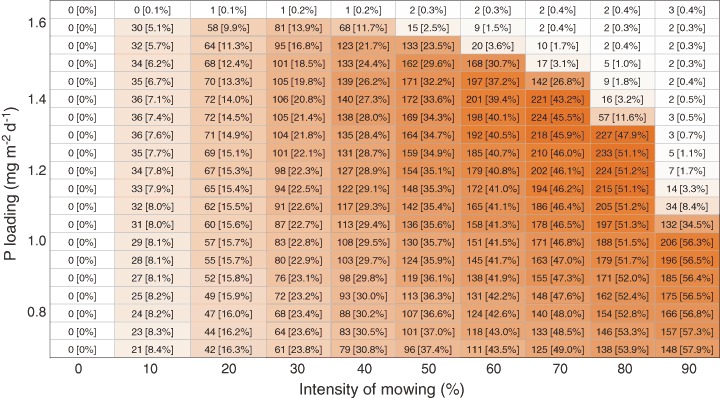
The amount of P (mg m^−2^ year^−1^) extracted from the system via harvesting of plant biomass during the last year of mowing, for different combinations of external nutrient loading and mowing intensity. The *color* indicates the quantity. The relative removal, that is, the ratio of P in the harvested biomass to the total amount of P added to the system via external loading, is presented between squared brackets (%)

The amount of P harvested from the lake during a mowing event increases with mowing intensity and nutrient loading, and is highest close to the point where mowing leads to a regime shift, reaching a maximum of almost 230 mg P m^−2^ (Fig. 6). The relative removal of P increases with mowing intensity and can be as high as 58%. However, the relative removal decreases with increasing nutrient loading. The associated dry-weight of the harvested plant biomass is presented in Online Resource 3.

### Prolonged Mowing and Resilience

**Fig. 7:**
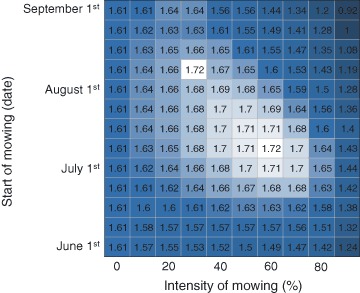
Effect of prolonged (long term) mowing on the CNL_eu_ (mg P m^−2^ day^−1^), i.e. the amount of nutrient input the lake can withstand without shifting to the turbid water state, for different combinations of mowing intensity and timing (start of the mowing procedure). The *colors* indicate whether mowing leads to an increase (*white*) or decrease (*dark gray*) of the critical nutrient loading (default 1.61 mg m^−2^ day^−1^)

Our model exercises show that in the long run repeated mowing is able to enhance the resilience of the clear water state to nutrient loading for a wide range of mowing intensities and mowing dates, as it leads to an increase (max. 7%) of the critical nutrient loading (CNL_eu_ > 1.61 mg P m^−2^ day^−1^; Fig. 7). Mowing during July and August in combination with an intermediate mowing intensity is most beneficial for enhancing the CNL_eu_. Mowing in early-summer or in late-summer can lead to a reduced resilience to nutrient loading (CNL_eu_ < 1.61 mg P m^−2^ day^−1^).

## Discussion

### Temporal Relief of Nuisance

Our modeling study shows that mowing can result in a temporal reduction of plant cover for a range of nutrient loadings and mowing intensities. These reductions of plant cover can reduce nuisance for up to several weeks, especially when the mowing intensity is fairly high and the external nutrient loading is low or moderate. Our model thus indicates that mowing can facilitate multiusage of shallow lake ecosystems. At lower intensities mowing also reduces the summer average plant cover, but this may be not sufficient to actually reduce nuisance as the remaining cover still exceeded the threshold level, which we fixed at 40%. Our model analyses indicate that it becomes more difficult to design a convenient mowing strategy when the external nutrient loading is high; the attraction of the alternative equilibrium is so strong that a rather small reduction in plant volume may be sufficient to trigger a shift to phytoplankton dominance. Interestingly, our results elucidate that a reduction of external nutrient loading alone is not an effective measure to drive back nuisance caused by aquatic plants (Fig. 4), which emphasizes the need for mowing. Because the risk of inducing a regime shift by mowing increases with external nutrient loading, the successfulness of mowing to reduce macrophyte nuisance goes hand in hand with the reduction of external nutrient loading. At what percentage of cover lake users perceive plants as a nuisance will vary between lakes and types of users. We took 40% because this number is frequently used in the gray literature, mostly in relation to growth of largemouth bass—a popular target species for sport fisheries (e.g. Gettys et al. 2014). However, we can hypothesize that when lake users already perceive nuisance at a lower plant cover (<40%) it will become increasingly difficult, or even impossible, to manage the vegetation successfully by harvesting while maintaining clear water. Vice versa, if local lake users would be more tolerant to the aquatic vegetation and perceive nuisance at higher percent cover (>40%), it will be more easy to reduce nuisance and maintain a clear-water ecosystem, especially when the external nutrient loading is not close to the critical nutrient loading level (Fig. 4). Hence, before designing a management scheme it is important to identify which stakeholders need to be served and at what percentage of plant cover they actually perceive plants as a nuisance.

### The Importance of Timing

Our model analyses indicate that the highest reductions of plant biomass can be achieved by mowing in mid-summer, while mowing in late summer appears to be least recommendable. The latter is not just because the peak of the holiday season (and thus recreational usage) is in mid-summer, but also because the risk of inducing a regime shift increases when mowing is conducted later in the growing season. In our model, mowing late in the growing season provides the aquatic plants with little opportunity to regain biomass before the growing season ends. This is in line with Engel (1990), who observed slow regrowth after mowing in July compared to mowing in June, and ascribed this to declining day length and water temperature. Consequently, in the following spring the macrophytes may start the competition with phytoplankton on their back foot, which eases a shift to phytoplankton dominance (Scheffer 2004, p. 280). Mowing too early in the growing season also bears a certain risk of triggering a regime shift, as our study showed, particularly when the external nutrient loading is high. We hypothesize that this is because the inter-specific competition with phytoplankton in early June is still rather strong, and setting back the submerged macrophytes favors phytoplankton growth. At the peak of the growing season, on the other hand, the intra-specific competition among macrophytes becomes more controlling, and mowing reliefs this intraspecific competition. Hence, the net growth rate of the macrophytes directly after mowing relates positively to mowing intensity (e.g. Fig. 2: the net growth rate after 30 and 60% mowing is 0.017 and 0.022 day^−1^, respectively). This compensatory growth is not sufficient however to compensate for the entire loss of biomass, as plant cover does not recover to pre-harvesting levels (Fig. 2).

The effect of timing on the impact of mowing may be different in field situations, particularly when the macrophyte community comprises growth forms that—unlike e.g. *Elodea canadensis*—produce overwintering organs (Scheffer 2004, p. 279). Hence, in case of propagule forming macrophyte species such as several *Potamogeton* and *Myriophyllum* species, these propagules may have already been formed when harvesting takes place late in the growing season, wherefore the impact on the next growing season is much smaller. Harvesting earlier in the season would then be an effective way to reduce the potential for macrophyte plant growth in the succeeding year, as that would prevent the formation of propagules (Wade 1990). Interestingly, a reduction of plant volume in the succeeding year is generally considered as a positive result of harvesting (e.g. Dall’Armellina et al. 1996), while our modeling study hints that this strategy is not without risks when lakes have alternative stable states and the external nutrient loading is high.

### Restrictions to Harvesting

In our model study we harvested fractions of the macrophytes to levels that may be unfeasible in real field situations. For example, there are practical reasons which frustrate harvesting large quantities of aquatic plants, as it is a labor-intensive and expensive activity. A simple calculation learns that for our modeled (circular) lake with a diameter of 1000 m, when receiving 1.2 mg P m^−2^ day^−1^, a harvesting intensity of 80% implies removing more than 650 tons of fresh biomass in just a short time span, assuming a fresh-weight:dry-weight ratio of 10 (e.g. Boiché et al. 2011; Dorenbosch and Bakker 2011; Online Resource 3). Secondly, local laws and regulations, such as the Dutch flora and fauna law, may impose restrictions on harvesting intensity and timing. Plants provide habitat and food for many species and it has been reported that significant amounts of fish and macroinvertebrates are removed along with the plants during harvesting (Engel 1990), which may include protected species. Furthermore, removing large quantities of plants may conflict with the protection of waterbirds that feed on the plants or the fauna living in macrophyte beds. A third reason is that in a field situation it will always be difficult to estimate the amount of aquatic plants that should be present to safeguard a clear water state, forcing lake managers to take a conservative approach when designing their plans. Hence, even though a submerged plant cover as low as 20% may coincide with good water quality (e.g. Portielje and Van der Molen 1998; Yanran et al. 2012), Hilt et al. (2006) advise to take 50% vegetation cover as a rule of thumb, and suggest that remaining stands after harvesting should still cover 50% of the lake. Also the Dutch authorities advise to remove maximally 50% of the plant cover, and even suggests to mow only 10% in case of native plant species (Rijkswaterstaat 2012). In our study we used *Elodea* sps. as model macrophytes, which are invasive in Europe. Our results show that part of the macrophytes should be retained under mowing management to prevent phytoplankton blooms under more eutrophic conditions. Implicitly this suggests that non-native macrophytes may be able to fulfill some of the ecosystem functions of native submerged macrophytes, in this case maintaining water clarity (Carpenter and Lodge 1986). This is in line with recent findings that non-native macrophytes may fulfill ecosystem functions similarly to their native counterparts and that their effectiveness depends rather on species traits than their origin (Grutters et al. 2015, 2016). Hence in management, complete removal of non-native macrophytes may be counterproductive for the ecosystem, if there are no native macrophytes to fill the empty place (Hussner et al. 2016).

### Spatial Heterogeneity

From our analyses it appears that harvesting 10% of the standing crop has only a marginal effect on reducing nuisance. This situation may change however when it is possible and desirable to spatially divide functions over the lake area. By harvesting in such a way that only certain patches are cleared, it may become possible to reduce nuisance locally e.g. in a zone designated for swimming or a channel for navigation. The model we used (PCLake) is not spatially explicit and is therefore not suited to evaluate the effect of a local disturbance by harvesting, as it is intended to provide a general indication of the harvesting pressure the lake can withstand. There is only little known about the effect of spatial heterogeneity on alternative stable states in shallow lakes. Theoretical studies suggest that the potential of local disturbances to instigate an ecosystem-wide regime shift increases with interconnectedness (dispersion) within the system (Van Nes and Scheffer 2005), and decreases with spatial heterogeneity (Van de Leemput et al. 2015). These studies thus suggest that alternative stable states are unlikely to persist side by side in lakes which are very homogenous. This means that local mowing becomes risky as over-harvesting has catastrophic consequences for the entire lake, albeit the regime shift may be gradual (Bel et al. 2012; Van de Leemput et al. 2015). When lakes do exhibit spatial heterogeneity e.g. in terms of depth, fetch or sediment composition, the response to a local perturbation becomes much more difficult to predict (Van de Leemput et al. 2015), but this heterogeneity can potentially lead to coexistence of contrasting states. The latter would create opportunities for localized harvesting practices. A follow up step is to couple the ecological modules of PCLake to 2D-hydrodynamic models to analyze harvesting in a spatial hydrodynamic context. This development is still in its infancy however (e.g. Van Gerven et al. 2015).

### Collateral Effects

Generally, not all cut plant biomass is removed from the lake due to inefficiency of the harvesting equipment (Hussner et al. 2016). The fragments that are not collected start to decompose in the water column, thereby releasing nutrients and contributing to the depletion of oxygen which in turn can stimulate internal nutrient loading from the sediment (Hilt et al. 2006). Additionally, cutting machinery may cause resuspension of sediments, which may reduce transparency and stimulate nutrient recycling. These side-effects of mowing are expected to be detrimental to ecosystem functioning (Rijkswaterstaat 2012), but it is difficult to quantify their true importance in the field. For simplicity reasons, we did not consider the effect of collateral disturbance in our main analyses. Yet, we did briefly look into their relative importance (presented as Online Resource 2), which revealed that, for the modeled circumstances and assumptions, the effect of collateral damage is marginal. This finding is in line with Carpenter and Gasith (1978) who reported short lived or insignificant effects on the littoral environment after clearing a 0.2 ha patch. Only when a regime shift has already been initiated, our model shows that the collateral effects of mowing stimulate the upheaval (Online Resource 2). However, we did not consider all potential side effects of mowing invasive aquatic macrophytes. For example, a factor we did not consider in this study is that many nuisance species (including *Elodea* spp.) spread by vegetative fragmentation (Hilt et al. 2006; Redekop et al. 2016). Mowing can stimulate dispersal of non-native nuisance species when fragments are produced that easily ride with the flow and settle at new places (Abernethy et al. 1996; Zehnsdorf et al. 2015). Especially when surrounding lakes or waterways are still free of these exotics, the vegetative dispersal capacity of the nuisance species that is being managed should be taken into consideration (Zehnsdorf et al. 2015). Recently, Hussner et al. (2016) reviewed how management aimed at the reduction or eradication of invasive aquatic plants can impact other (native) species present in the ecosystem. Interestingly, these effects can be both positive and negative. For example, Dawson et al. (1991) reported a case where 30 macroinvertebrate individuals were removed per gram dry weight of cut aquatic plants, while Bickel and Closs (2009) showed that total invertebrate biomass and abundance was significantly higher in the areas where mowing took place compared to the untreated macrophyte beds. Moreover, while Engel (1990) reported that up to 450 fish were removed per 100 kg fresh weight of cut aquatic plants, the potential for improving growth and size structure of fishes by reducing macrophyte density has long been recognized (e.g. Wiley et al. 1984; Olson et al. 1998). Furthermore, vegetation is a major food source for many waterfowl species aquatic and it is known that herbivorous birds such as coots (*Fulica*) can have a large impact on vegetation density (Van Altena et al. 2016). Interestingly, this trophic interaction may give rise to an interaction effect between mowing and herbivory. Hence, if a large quantity of vegetation is removed by means of mechanical mowing, this may either cause waterfowl to leave, but it may also cause birds to put extra pressure on the remaining vegetation, potentially triggering a critical regime shift to the turbid state (Van Altena et al. 2016).

### Removal and Recovery of Nutrients

Because there are nutrients stored in plant tissue, as well as in material attached to the plant surface such as periphyton and calcite incrustations, the removal of submerged macrophytes may help to remediate the detrimental effects of eutrophication, both in the lake where the plants are removed from, and in downstream aquatic ecosystems (Carpenter and Adams 1977). Our modeling scenarios indicate that the highest amount of P is extracted from the system when both the external P loading and the mowing intensity are high. The relative removal however, which tells more about the capacity to actually prevent further enrichment of the system via harvesting, increases with decreasing nutrient loading, maximally reaching 58% in our analyses. While it should be noted that periphyton and calcite incrustations are not explicitly modeled by PCLake, we find these numbers to be grossly in line with estimations presented in the literature. For example, for a eutrophic lake with 30% plant cover, Carpenter and Adams (1977) estimated that a relative removal of 37% of the P loading could be established if all plants would be harvested. Conyers and Cooke (1983) reported that a relative removal of 44% could be reached in a mesotrophic lake with 43% plant cover. Moreover, Wile (1978) presented a case where harvesting operations resulted in the removal of 560 kg P, and estimated the relative removal to be 47%. It is important to realize however that these numbers refer to the relative removal of P from the system as a whole, and not solely from the water column (Burton et al. 1978). Although many rooted macrophytes species are well capable of assimilating nutrients directly from the water column through their shoots (Madsen and Cedergreen 2002; Angelstein and Schubert 2008; Christiansen et al. 2016), at least part of their nutrients may be obtained from the sediment, especially in systems where large amounts of P are available in the sediment. As a consequence, removal of plant biomass does not axiomatically offset the external loading of P into the water column, and hence the incoming nutrients may perpetuate eutrophication. We postulate that the effect of harvesting rooted macrophytes on ecosystem functioning is highest when the macrophytes take up most of their nutrient directly from the water column. Furthermore, while harvesting alone may not be able to completely offset the incoming nutrient fluxes (Burton et al. 1978), we argue that the usefulness may be enhanced by the joint application of complementary management measures, such the application of phosphorus adsorbing natural soil and modified clay.

Harvested plant tissue can potentially serve as a source of nutrients, instead of only being waste material. The use of aquatic plant biomass to fertilize agriculture fields is an old practice (Roger and Watanabe 1984), which is still carried out in many parts of mainly the developing world. Recently harvesting aquatic plant biomass has been put forward as a way to close the P cycle (Quilliam et al. 2015). Although excessive growth of macrophytes indicates a local surplus of nutrients, P is a scarce element in many places, leading to phosphate starvation in crops, and global phosphate sources are declining rapidly (Cordell et al. 2009; Childers et al. 2011). The recovery of valuable P thus has the potential to increase the viability of harvesting as a management measure, which is otherwise a costly procedure (Hilt et al. 2006). Currently there is no agreement on how to maximize P uptake and removal by macrophytes (Quilliam et al. 2015). Our model results suggest that it is beneficial for lake managers to reduce the external nutrient loading as much as possible, as that will reduce the possibility of harvesting triggering an unwanted regime shift to a state without macrophytes, and increase the relative removal of P.

### Prolonged Harvesting

Model analysis of the long-term effects of harvesting suggests that harvesting can potentially be used to prevent nutrient over-enrichment by increasing the resilience of the system to external loading, that is, by increasing the CNL_eu_ (Fig. 7). It is important to note however that in this analysis harvesting was executed every year, and that we started off with a clear and oligotrophic lake—in the domain where no alternative state is apparent. Because of the latter, almost all macrophytes can be removed at the start of the analysis without risking a shift to the alternative state, as there simply is none. In turn, the removal of macrophytes prevents the accumulation of nutrients in the system, postponing the formation of an alternative equilibrium and hence increasing the CNL_eu_. This implies that the history of the lake is an important factor to consider when designing a mowing strategy. If nutrients have been able to accumulate in the lake prior to the mowing activities, as in our first analyses, the resilience of the lake to perturbations such as mowing may have already decreased and fairly small fractions of macrophyte removal may be enough to instigate a regime shift (see Online Resource 4 for an illustrative example). Thus, based on the long term mowing scenarios we argue two points. The first is that phytoremediation can be a worthwhile measure to prevent a lake from becoming eutrophic when it is still oligotrophic and its capacity to withstand perturbations is still high. The second is that it is much more difficult to use phytoremediation to impoverish a lake when it is already eutrophic, even though the absolute removal of nutrients is high, because the capacity to withstand perturbation is much reduced. As many vegetated lakes in NW Europe have only recently recovered from the turbid state, and their sediments are likely to be saturated with nutrients, mowing schemes should be designed with great care.

### From Model to Practice

An important question is how the results of this theoretical exercise should be interpreted by managers and can be useful in contemporary ecosystem management. Our point of departure is that every water system is unique (*n* = 1), but that there are general mechanisms that are key to the ecological functioning of every lake. PCLake has been developed to include the most important biotic and abiotic processes and lake characteristics (Janse et al. 2008). Moreover, to strive for generality, the model has been calibrated with data from >40 lakes with the aim to get the best overall fit (Janse et al. 2010). Hence, PCLake provides a coherent framework to investigate the effect of mowing within an ecosystem context with alternative stable states, allowing us to focus on important aspects of mowing, such as the intensity and timing, while keeping other factors constant. An important purpose of such analysis is to provide scientists and managers with working hypothesis about the way ecosystems function, and to contribute to the development of theory. The insights that are obtained by simulations cannot easily be derived from any other type of study, as the analyses would be too costly or unethical to do in natural systems. As such, these insights complement the insights obtained by alternative approaches, such as lab experiments and field observations (Peck 2004; Scheffer 2004, p. 313). PCLake is one of the very few integrated ecosystem models available for this kind of simulations (Janssen et al. 2015)

On one hand, we argue that the insights obtained by our simulations are widely applicable, as the model is built up of many general prevailing processes and principles. For example, although *Elodea* is used as a model species, we expect that, at least in qualitative terms, the response of other yet similar submerged angiosperms, such as *Lagarosiphon major* or *Egeria densa*, will be comparable. Moreover, lake characteristics have been chosen such that the model describes a hypothetical lake that is representative for many small and shallow lakes in the temperate zone. Interestingly, PCLake has even shown to be useful outside the temperate zone (e.g. Mellios et al. 2015; Kong et al. 2016).

On the other hand we acknowledge that the results of PCLake are highly dependent on the lake characteristics modeled. For example, Janse et al. (2008) showed that the resilience of shallow lakes decreases with increasing depth and fetch, implying that in larger and deeper lakes mowing can more readily result in a regime shift to the phytoplankton-dominated turbid state. Also the choice for a specific threshold level where lake users perceive plants as nuisance has implications for our results. When for a given case study, these controlling factors deviate too much from the settings used in this study, the calculations presented here should be redone for the new setting. Please note that such limitations apply to any type of experiment focusing on few independent variables.

Of course, it is conceivable that the ecology of certain lakes may differ fundamentally from the system currently portrayed by PCLake. For example, Blindow et al. (2014) distinguish between a charophyte-dominated clear water state and an angiosperm-dominated clear water state, and report on notable differences in the strengths of the exerted positive feedback loops with water clarity. Effects may be even more profound when a certain process has a strong effect on the functioning of a specific lake, but is not covered by the model. For example, grass carp (*Ctenopharyngodon idella*) has a strong trophic interaction with aquatic plants (Hussner et al. 2016), however this fish species is currently not included in PCLake. In all cases, a customized PCLake study, whereby the model is adapted, calibrated and validated for a specific case, will provide the most accurate predictions which can be readily employed in ecosystem management (e.g. Witteveen+Bos 2010; Nielsen et al. 2014; Trolle et al. 2014; Kong et al. 2016). The present study provides a clear example of how to set up a model analysis with PCLake to evaluate the effect of mowing on shallow lake ecosystem functioning.

## Conclusions

Our integrated modeling analysis of a typical shallow lake in the temperate zone indicates that harvesting submerged macrophytes can be effective in temporarily reducing nuisance in lakes which are oligo—or mesotrophic, particularly when mowing is executed in mid-summer. Designing a successful mowing strategy becomes less easy with increasing nutrient loading. More eutrophic lakes are less resilient to perturbations, and when the external nutrient loading approaches the critical level, relatively small reductions in plant cover are sufficient to trigger an unwanted shift to the alternative phytoplankton dominated state. By extracting nutrients from the lake, negative effects of eutrophication may be partially remediated. Our modeling indicates that the largest amounts of P can be recovered close to the tipping point, although the highest removal of P relative to the input of P is realized when the external P loading is low. Particularly when a lake is still oligotrophic, phytoremediation can be an effective measure to counteract slowly increasing nutrient inputs, while it appears more difficult to use harvesting to impoverish a lake which is already eutrophic, as more eutrophic lakes are also more sensitive to perturbations. These insights provide a basis for more tailored studies on the effects of harvesting in specific lakes systems.

## Electronic supplementary material

Supplementary Information
