# Supplementary material for: Mowing Submerged Macrophytes in Shallow Lakes with Alternative Stable States: Battling the Good Guys?
Source: Environ Manage. 2017 Jan 2;59(4):619–34. doi: 10.1007/s00267-016-0811-2 (PMC5339322; doi:10.1007/s00267-016-0811-2)
Supplement: Supplementary file 1 — Supplementary Information [file 267_2016_811_MOESM1_ESM.pdf]

Online Supplementary Material

Mowing submerged macrophytes in shallow lakes with alternative stable states: battling the good guys?

Jan J. Kuiper[1,2*], Michiel J.J.M. Verhofstad[1], Evelien L.M. Louwers[1,3,4], Elisabeth S. Bakker[1], Robert J. Brederveld[3], Luuk P.A. van Gerven[1,2], Annette B.G. Janssen[1,2], Jeroen M. de Klein[2] & Wolf M. Mooij[1,2]

1 Department of Aquatic Ecology, Netherlands Institute of Ecology, P.O. Box 50, 6700 AB Wageningen, the Netherlands; 2 Aquatic Ecology and Water Quality Management Group, Department of Environmental Sciences, Wageningen University, P.O. Box 47, 6700 AA Wageningen, the Netherlands; 3 Witteveen+Bos, P.O. Box 233, 7400 AV Deventer, The Netherlands; 4 Ecology & Biodiversity, Institute of Environmental Biology, Utrecht University, P.O. Box 80.084, 3508 TB Utrecht, the Netherlands

* Corresponding Author: j.kuiper@nioo.knaw.nl

This document contains:

- Online Resource 1 - Schematic representation of PCLake
- Online Resource 2 - The importance of collateral disturbance
- Online Resource 3 - Harvested biomass (dry-weight)
- Online Resource 4 - Long term vs. short term effects of mowing

Online Resource 1

Schematic representation of PCLake

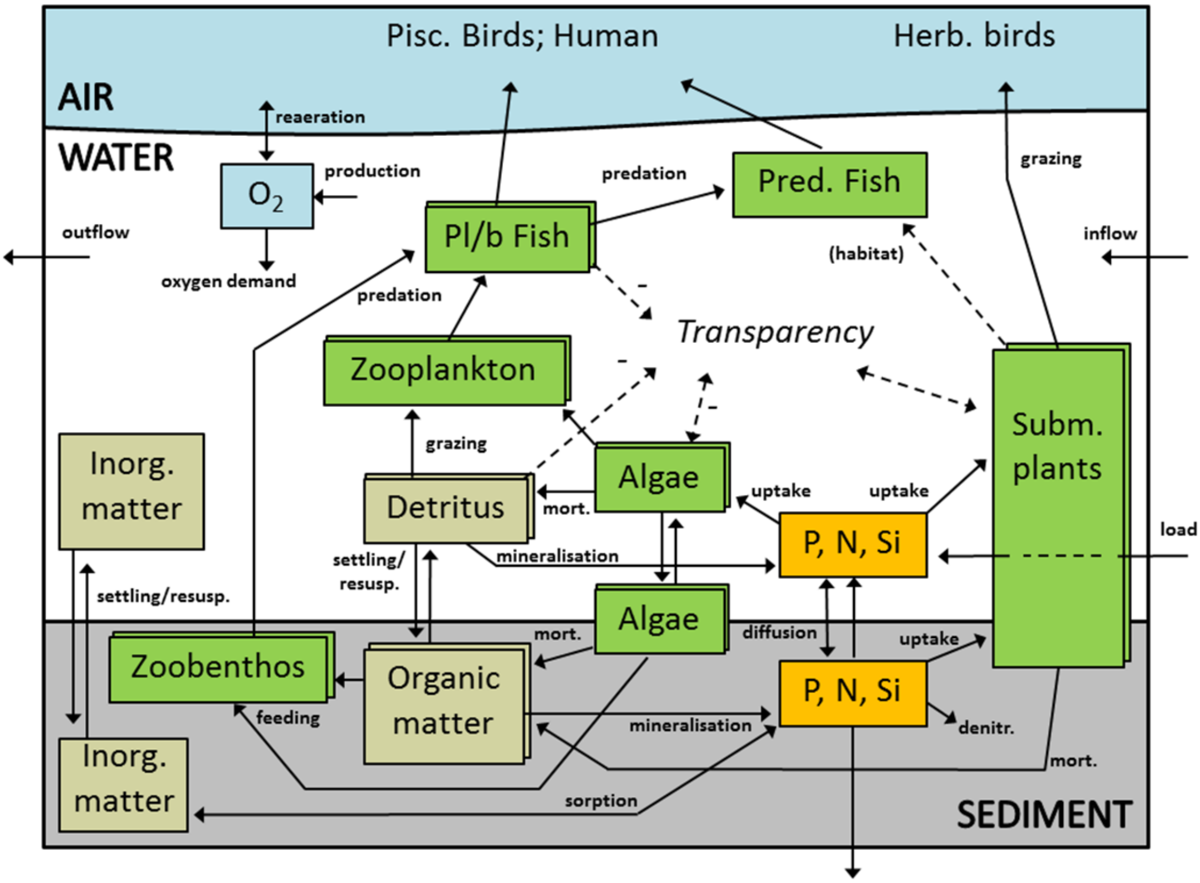

Figure S1. Schematic view of the structure of PCLake. Blocks denote the state variables of the model. Shaded blocks denote compartments modelled in dry weight, phosphorus and nitrogen (and silica in case of diatoms). Arrows denote mass fluxes. Respiration fluxes are not shown. Dotted arrows denote 'empirical' relations. The biota in PCLake are modeled as functional groups. The submerged macrophytes are assumed to be homogeneously distributed over the complete water column and are rooted in the sediment. Other groups in the water column are phytoplankton (three groups: 'diatoms', 'green algae' and 'cyanobacteria'), zooplankton, planktivorous fish, benthivorous fish and piscivorous fish. The biotic groups in the upper layer of the sediment include the zoobenthos and the settled fractions of the three types of phytoplankton. The abiotic components in the water column and in the sediment are detritus, inorganic material, dissolved phosphorus, ammonium, and nitrate. A full description of the model is presented by Janse (2005). The figure is modified after Janse (1997).

References

Janse JH (1997) A model of nutrient dynamics in shallow lakes in relation to multiple stable states. Hydrobiologia 342/343:1–8.

Janse JH (2005) Model studies on the eutrophication of shallow lakes and ditches. PhD Thesis. Wageningen University

Online Resource 2

The importance of collateral disturbance

Although the aim of water managers is to remove cut biomass from the water, part of the plant material is often left in the water due to inefficiency of the cutting machinery. This plant material in the water leads to increased light attenuation and stimulates nutrient recycling, disfavoring the growth of the remaining submerged water plants. Another factor that is potentially detrimental to the remaining vegetation is temporarily enhanced resuspension caused by the mowing procedure. This can for example result from thrust engines on mowing boats that stir up the sediment, or because roots are pulled out from the sediment during the cutting.

In the default version of PCLake only ‘clean’ mowing is considered, whereby all the mown biomass is removed from the system, without additional resuspension. Therefore, we modified the PCLake model equations in such a way that a defined fraction of the clippings remains in the system as detritus. We estimated the fraction to be 20%. Analogous to the detritus resulting from natural mortality, the largest share of this plant material (90%) sinks to the bottom to become part of the detritus pool in the sediment (Janse 2005). Furthermore, we developed a function that causes the resuspension of the sediment to increase linearly with mowing intensity, maximally reaching an additional 5 g m^{-2} d^{-1} of resuspended material, which is about 2.5 times the amount of sediment that is on average resuspended due to benthivorous fish in a turbid lake (Janse 2005 p.291). The resuspension is only enhanced during the mowing period. We analyzed the effects of these collateral disturbances on the within-season dynamics of the vegetation cover and chlorophyll-*a* for two different mowing intensities (60 and 90% respectively), an intermediate nutrient loading (1.3 mg P m^{-2} d^{-1}) and a single mowing date (July 1st). We compared the results with the default simulations without collateral disturbance caused by mowing.

For the used parameter settings, this analysis reveal no clear sign of collateral damage (Fig. S2a-d). According to our model, enhanced resuspension and remaining of plant material in the water column has a negligible effect when 60% of the submerged plants is cut (Fig. S2a,b), and this is still the case for a mowing intensity of 80% (results not shown). Only when the mowing activity instigates a regime shift, which is the case for a mowing intensity of 90%, the modelled collateral disturbances speed up the regime shift (Fig. S2c,d). Particularly the enhanced resuspension propels the lake faster to the alternative state. A more elaborated (sensitivity) analysis is needed to elucidate the importance of collateral disturbance. Important to note here is that we did not consider the dispersal aspect of plant fragments generated by the mowing action: vegetative plant fragments can easily spread with the water flow, potentially contributing to new invasions of waterweed in uninhabited waters (Zehnsdorf et al. 2015). This factor should be taken into consideration by water managers dealing with exotic species.

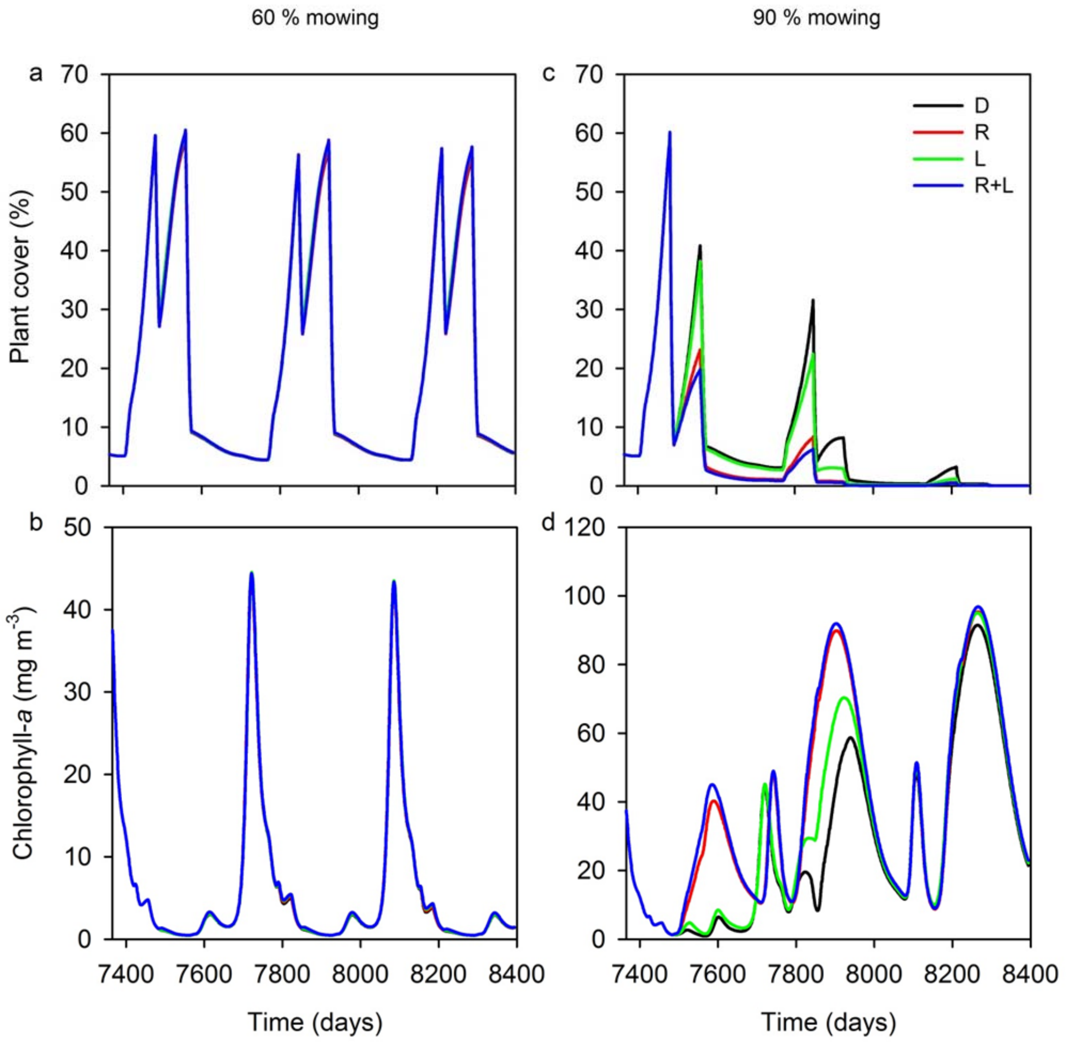

Figure S2. Effects of collateral disturbance caused by the mowing procedure on July 1st the dynamics of plants and phytoplankton in three succeeding years for a lake receiving 1.3 mg P m^{-2} day^{-1}, for 60 percent mowing and 90 % mowing. D is default (black line), R is enhanced resuspension (red line), L is leaving 20 % of the mowed plant biomass in the water column (green line) and R+D is a combination of the latter two (blue line).

References

Janse JH (2005) Model studies on the eutrophication of shallow lakes and ditches. PhD Thesis. Wageningen University

Zehnsdorf A, Hussner A, Eismann F, et al (2015) Management options of invasive Elodea nuttallii and Elodea canadensis. Limnol - Ecol Manag Inl Waters 51:110–117. doi: 10.1016/j.limno.2014.12.010

Online resource 3

Harvested biomass (dry-weight)

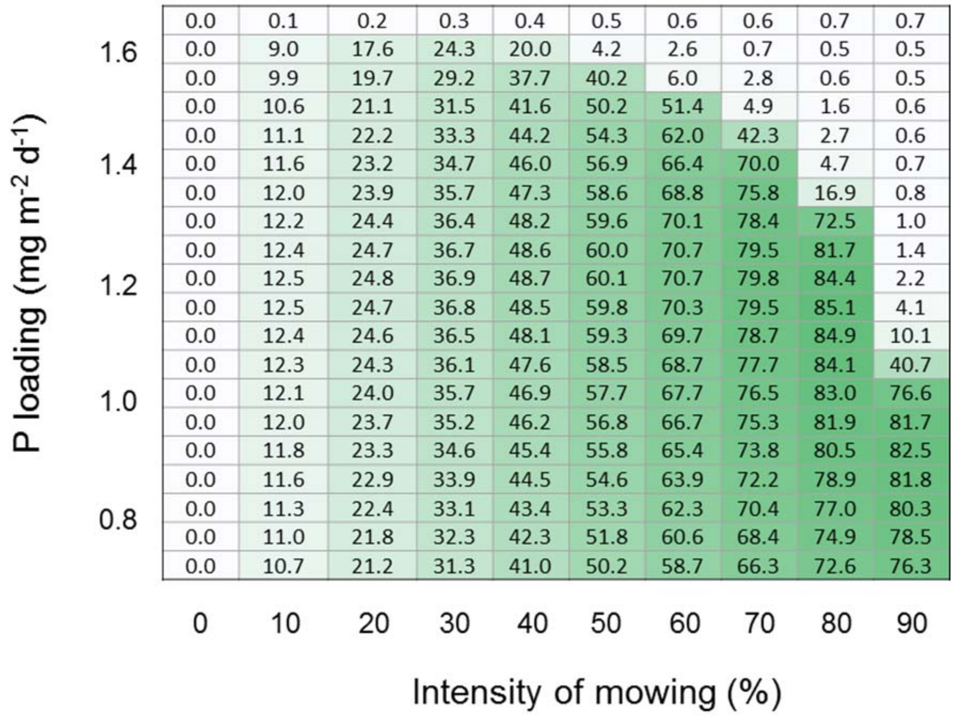

0	10	20	30	40	50	60	70	80	90
0.0	0.1	0.2	0.3	0.4	0.5	0.6	0.6	0.7	0.7
0.0	9.0	17.6	24.3	20.0	4.2	2.6	0.7	0.5	0.5
0.0	9.9	19.7	29.2	37.7	40.2	6.0	2.8	0.6	0.5
0.0	10.6	21.1	31.5	41.6	50.2	51.4	4.9	1.6	0.6
0.0	11.1	22.2	33.3	44.2	54.3	62.0	42.3	2.7	0.6
0.0	11.6	23.2	34.7	46.0	56.9	66.4	70.0	4.7	0.7
0.0	12.0	23.9	35.7	47.3	58.6	68.8	75.8	16.9	0.8
0.0	12.2	24.4	36.4	48.2	59.6	70.1	78.4	72.5	1.0
0.0	12.4	24.7	36.7	48.6	60.0	70.7	79.5	81.7	1.4
0.0	12.5	24.8	36.9	48.7	60.1	70.7	79.8	84.4	2.2
0.0	12.5	24.7	36.8	48.5	59.8	70.3	79.5	85.1	4.1
0.0	12.4	24.6	36.5	48.1	59.3	69.7	78.7	84.9	10.1
0.0	12.3	24.3	36.1	47.6	58.5	68.7	77.7	84.1	40.7
0.0	12.1	24.0	35.7	46.9	57.7	67.7	76.5	83.0	76.6
0.0	12.0	23.7	35.2	46.2	56.8	66.7	75.3	81.9	81.7
0.0	11.8	23.3	34.6	45.4	55.8	65.4	73.8	80.5	82.5
0.0	11.6	22.9	33.9	44.5	54.6	63.9	72.2	78.9	81.8
0.0	11.3	22.4	33.1	43.4	53.3	62.3	70.4	77.0	80.3
0.0	11.0	21.8	32.3	42.3	51.8	60.6	68.4	74.9	78.5
0.0	10.7	21.2	31.3	41.0	50.2	58.7	66.3	72.6	76.3

Figure S3. Amount of vegetation dry weight biomass that is harvested from the system (g m^{-2} $year^{-1}$). The green color indicates the quantity.

Online Resource 4

Long term vs. short term effects of mowing

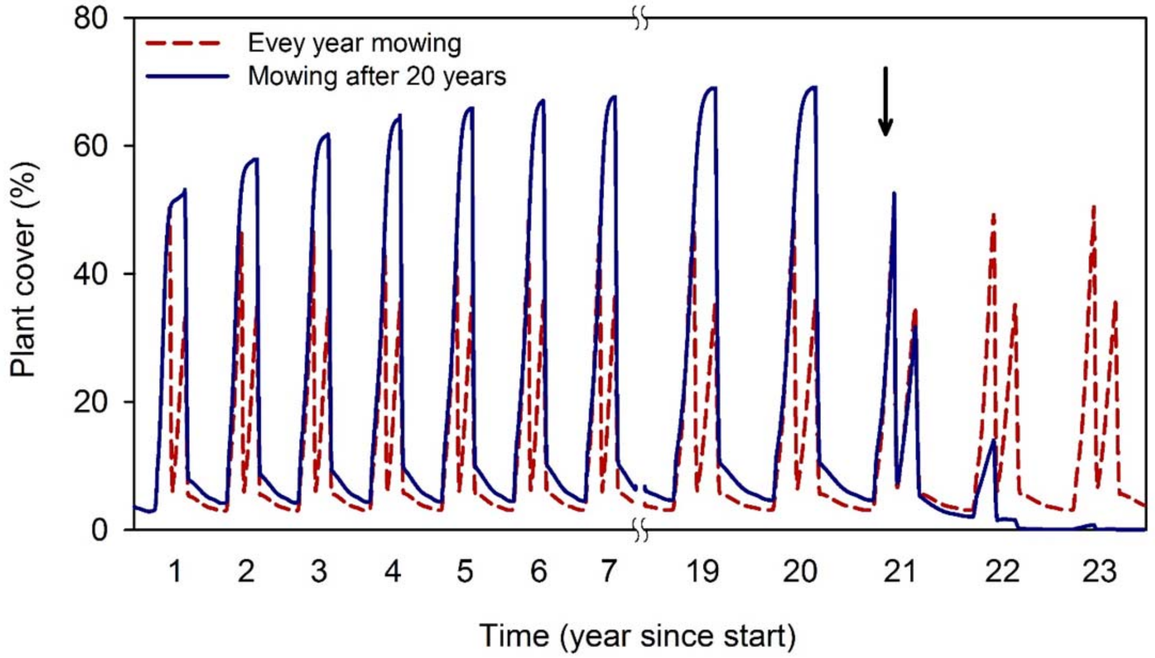

Figure S4. We modelled a lake receiving 1.2 mg P m^{-2} day^{-1} and applied a mowing intensity of 80%. The only difference between the two scenario's is that in one scenario mowing starts right from the beginning, while in the other scenario mowing starts after twenty years. When mowing is applied directly from the start, the systems moves to an equilibrium situation in which large reductions in plant cover (80%) can be achieved (red striped line). When in the first twenty years no mowing is applied, the system goes to a different equilibrium: after twenty years, when mowing is applied for the first time, the same mowing intensity (80%) instigates a regime shift to the turbid state (blue line). Hence, in the first twenty years nutrients have been able to accumulate in the lake, which lowered the resilience of the lake to perturbations such as mowing.
